# Allergic Diseases Caused by *Aspergillus* Species in Patients with Cystic Fibrosis

**DOI:** 10.3390/antibiotics10040357

**Published:** 2021-03-28

**Authors:** Aidan K. Curran, David L. Hava

**Affiliations:** 1 Pulmatrix Inc., 99 Hayden Avenue, Lexington, MA 02421, USA; acurran@pulmatrix.com; 2 Synlogic Inc., 301 Binney Street, Cambridge, MA 02142, USA

**Keywords:** allergic bronchopulmonary aspergillosis, cystic fibrosis, anti-fungal, itraconazole

## Abstract

*Aspergillus* spp. are spore forming molds; a subset of which are clinically relevant to humans and can cause significant morbidity and mortality. *A. fumigatus* causes chronic infection in patients with chronic lung disease such as asthma, chronic obstructive pulmonary disease (COPD) and cystic fibrosis (CF). In patients with CF, *A. fumigatus* infection can lead to allergic disease, such as allergic bronchopulmonary aspergillosis (ABPA) which is associated with high rates of hospitalizations for acute exacerbations and lower lung function. ABPA results from T_H_2 immune response to *Aspergillus* antigens produced during hyphal growth, marked by high levels of IgE and eosinophil activation. Clinically, patients with ABPA experience difficulty breathing; exacerbations of disease and are at high risk for bronchiectasis and lung fibrosis. Oral corticosteroids are used to manage aspects of the inflammatory response and antifungal agents are used to reduce fungal burden and lower the exposure to fungal antigens. As the appreciation for the severity of fungal infections has grown, new therapies have emerged that aim to improve treatment and outcomes for patients with CF.

## 1. Pulmonary *Aspergillus* Infections

*Aspergillus spp.* are ubiquitous spore forming molds, a subset of which are clinically relevant to humans and can cause significant morbidity and mortality. Pulmonary infection from *A. fumigatus*, the most common *Aspergillus* pathogen, causes a diverse set of diseases, ranging from acute invasive disease to long-term, chronic infections [1]. The type of disease and disease severity are largely dictated by the immune system of the host. Immunosuppressed patients, such as those undergoing organ transplantation or cancer treatment, are at high risk for invasive aspergillosis (IPA). IPA is a life-threatening disease that occurs following the inhalation of fungal conidia and the evasion of host defense that allows the fungus to invade host tissues and grow unchecked in the lung [2]. The mortality rate of IPA can be as high as 90% in some patient populations [3,4] and prophylaxis using oral antifungal drugs is commonly used to prevent infections. Chronic pulmonary aspergillosis is distinct from IPA and manifests in a variety of different diseases including aspergilloma, cavitary disease and fibrosing disease [5]. Patients with chronic lung disease such as asthma, chronic obstructive pulmonary disease (COPD) and cystic fibrosis (CF) are susceptible to chronic aspergillosis. 

Chronic diseases caused by *Aspergillus* can result from stable active infection of the lung or from allergic sensitization resulting from the exposure to *Aspergillus* antigens. In the first case, disease results from stable and persistent infection of the airways with *Aspergillus* resulting in fungal growth and an inflammatory response that aims to clear the infection from the lung. In some instances, this has been referred to aspergillus bronchitis [6], which may have a varying impact on lung function and clinical disease. In contrast, allergic diseases, characterized by a T_H_2-driven immune response to *Aspergillus* antigens, include both severe asthma with fungal sensitization (SAFS) and allergic bronchopulmonary aspergillosis (ABPA). Both SAFS and ABPA are significant clinical issues in patients with asthma, with the latter being a significant clinical problem in patients with CF [7,8]. *A. fumigatus* is the common cause of ABPA, however sensitization to other *Aspergillus* species has been noted [9]. In the case of allergic disease, the resulting allergic response to antigen is likely independent of the specific *Aspergillus* species. It has been suggested that there may be a continuum of disease that starts with aspergillus bronchitis and progresses to sensitization and ultimately ABPA [6], for the purposes of this review, we primarily focus on aspergillus bronchitis and APBA in patients with CF.

**Figure 1:**
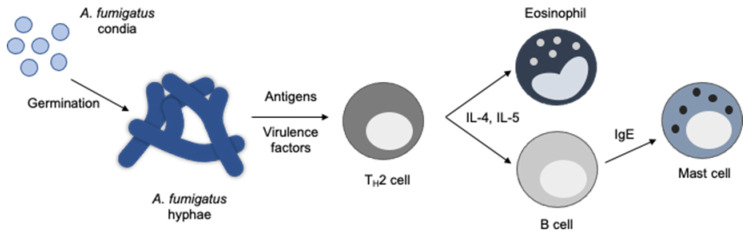
Pathophysiology of ABPA. Inhalation of fungal conidia and subsequent germination of fungal hyphae results in the expression of antigens specific to hyphal growth. These antigens are recognized by the immune system and cause a T_H_2 driven immune response marked by increased levels of T_H_2 cytokines, recruitment of eosinophils to the lung and increased production of IgE. The activation of eosinophils and mast cells drive the pathophysiology of ABPA and the resulting clinical symptoms.

While the clinical impact of *Aspergillus* colonization and persistence may vary among patients and require continued characterization, allergic fungal infections have a clear deleterious clinical impact. CF patients with sensitization to *A. fumigatus* antigens have a distinct and robust T_H_2 inflammatory response in sputum samples after allergen challenge. This inflammation is marked by increases in sputum eosinophils and increased expression of IL-5 and IL-13 [10]. ABPA is characterized by a complex T_H_2 hypersensitivity reaction in response to fungal antigens that drives immune cell activation and eosinophil recruitment (Figure 1) [11,12]. Expression of IL-4 and IL-5 are central to these processes. IL-4 stimulates the upregulation of adhesion molecules involved in eosinophil recruitment and the production of IgE by B cells, which in turn leads to mast cell activation. IL-5 produced by both T_H_2 cells and mast cells is a key mediator of eosinophil activation. Activation of both mast cells and eosinophils results in the release of mediators that induce bronchoconstriction (Figure 1) [12]. Through repeated cycles of inflammation, patients with ABPA are at high risk for frequent exacerbations and the development of bronchiectasis [1]. 

## 2. Prevalence and Diagnosis of *Aspergillus* Infections in Patients with CF

Chronic aspergillosis and ABPA affect a large number of patients each year, with more than 3 million cases of chronic disease and nearly 5 million cases of ABPA reported on an annual basis [7,13]. The majority of ABPA represents disease in asthmatics, with an estimated 1 to 2.5% of all asthmatics worldwide having ABPA [14]. Reports of ABPA prevalence in patients with CF vary from 1 to 15% [15,16], with reports of colonization rates in respiratory samples ranging from 6 to 58% [13,17,18]. The variability in these reports is likely at least partially due to differences in sample collection, processing and diagnostic approaches between laboratories. 

*A. fumigatus* is the most common species present in the lungs of patients with CF, however, other *Aspergillus* species are clinically relevant, including *A. niger, A. terrus* and *A. flavus* [15,19,20]. The prevalence of *Aspergillus* in adult CF patients has been appreciated for a number of years, with increased prevalence associated with prophylactic antibiotic use [21,22]. More recently, an increase in prevalence has been observed in young children with CF [23,24,25]. In infants with CF, *Aspergillus* spp. were detected in bronchoalveolar lavage (BAL) samples with similar prevalence and at similar ages as the common the bacterial colonizers *Staphylococcus aureus*, *Pseudomonas aeruginosa,* and *Haemophilus influenzae*. However, whereas *S. aureus* and *P. aeruginosa* prevalence decreased over time due to antibiotic therapy, *Aspergillus* prevalence remained unchanged [23]. 

An appreciation for the clinical significance of *Aspergillus* colonization and persistence has increased in recent years. Several studies have suggested an association between *A. fumigatus* infection, respiratory function and severe pulmonary exacerbations. In several studies, CF patients with chronic *A. fumigatus* infection have lower percent predicted forced expiratory volume (FEV_1_) than uninfected controls [17], higher rates of hospitalization resulting from pulmonary exacerbations [17], show more rapid loss of lung function [24,26,27] and have worse respiratory quality of life [28]. Declines in clinical disease are a result of increased pulmonary inflammation [29,30], which can also result in structural lung changes by high-resolution CT (HRCT) scan. In a cross-sectional study of children, BAL samples positive for *Aspergillus* were associated with air-trapping on HRCT, although the same study failed to show an association with lung function decline between the ages of 5 and 14 [31]. A recent longitudinal study of 330 children found similar findings linking *Aspergillus* culture positivity to changes in structural lung disease [30]. *Aspergillus* infection was associated with worse initial CT scores that declined further in the subsequent year, with the most significant impact on air-trapping and mucus plugging [30]. Interestingly, the magnitude of disease progression was associated with the number of *Aspergillus* infections over the course of the study, suggesting a dose-responsive relationship between infection and disease. A similar association with progression of lung diseases was observed for *P. aeruginosa* infection, but not for *S. aureus* and *H. influenzae* infections. 

An understanding of incidence and prevalence is further hampered by the difficulty of diagnosing disease. Diagnostic criteria for ABPA include both obligatory and supportive criteria in addition to having either asthma or CF. High levels of serum IgE (>1000 U/mL) and a positive hypersensitivity skin test or increased IgE antibody to *Aspergillus* are required criteria together with at least two additional supportive features: eosinophilia (>500 cells/μL), *Aspergillus*-specific IgG, and/or radiographic findings [32]. ABPA is characterized clinically by wheezing, dyspnea, mucus production and productive cough and bronchoconstriction. Repeated episodes of mucus production, bronchial obstruction and inflammation may lead to bronchiectasis and, in severe cases, pulmonary fibrosis, which collectively result in a progressive loss of lung function. A combination of clinical symptoms and biomarkers have been used to develop a staging system to help in disease management [32].

The range of reported disease prevalence and variations in diagnostic approaches suggests that pulmonary fungal infections in CF may be under diagnosed. This is further complicated by the use of culture-based diagnostic methods, which underestimate *Aspergillus* detection compared to molecular methods, such as quantitative PCR [6,33]. New methods to detect *Aspergillus* in sputum using RT-PCR and high-volume culture techniques have the potential to significantly increase the sensitivity of detection and ultimately, diagnosis [13,34]. Using these new culture techniques, CF patients can be put into four subgroups; those without aspergillosis, those sensitized to *Aspergillus*, those with ABPA and those with aspergillus bronchitis [13]. Using this methodology, Baxter et al. classified 130 CF patients and found that 30% had aspergillus bronchitis and 17.7% had ABPA. Armstead et al. compared these rates to the reported rates of ABPA in CF registries and literature reports for adult CF patients from 30 different countries [35]. They found that the number of ABPA cases diagnosed and reported is likely a significant underrepresentation of the estimated cases when more sensitive diagnostic assays are utilized. In the United States the number of documented adult CF cases of ABPA (869 cases) was 34.6% of the estimated cases (2510 cases) as defined by Armstead et al. Using the more recent data, almost 50% of US adult CF patients may have either ABPA or aspergillus bronchitis [35].

## 3. *Aspergillus* ssp. and Bacterial Interactions in the Pathogenesis of Disease

CF patients have a complex lung microbiota, where there is likely significant interplay between colonizing bacteria and fungi [36]. Longitudinal assessment of data from the Cystic Fibrosis Foundation Patient Registry suggests that *P. aeruginosa* and *Stenotrophomonas maltophilia* infections are positively correlated with *Aspergillus* infections [37]. In contrast, infection with *Burkholderia cepacia* complex was negatively associated with both current and future *Aspergillus* infection [37], indicating that there are specific mechanisms in different bacteria that influence the susceptibility of patients to *Aspergillus* infections. Potential interactions between *Aspergillus* species and non-tuberculous mycobacterial (NTM) infections have not been well characterized. Given the importance of NTM infections in CF [38] and the suggestion that *A. fumigatus* can negatively impact NTM infection in mice [39], a more detailed assessment of the impact of co-infection with these pathogens is needed. 

In CF patients, the most common bacterial and fungal isolates are *P. aeruginosa* and *A. fumigatus,* respectively [40] and colonization with both species results in greater lung function decline relative to individuals with either infection alone [17,41]. Given the relationship between bacteria and fungi, there is growing interest in understanding how these infections interact, influence each other and affect the progression of CF-related disease. In particular, it is important to understand how the treatment of one infection may increase or decrease susceptibility to another infection. Indeed, in clinical practice, antibiotic treatment has been shown to predispose CF patients to *Aspergillus* colonization [22]. 

Both *P. aeruginosa* and *A. fumigatus* form biofilms in vivo and in vitro [42,43]. Several reports suggest that *P. aeruginosa* inhibits *A. fumigatus* planktonic growth and biofilm formation by secreted factors and different isolates of *P. aeruginosa* exhibit different degrees of toxicity [41,44]. *P. aeruginosa* inhibits *A. fumigatus* growth by the production of pyoverdine, a siderophore that sequesters iron. *P. aeruginosa* mutants defective in pyoverdine production are not toxic to *A. fumigatus*, and the addition of pyoverdine to mutant cultures restores *A. fumigatus* toxicity [45]. More research will be required to understand not only the in vivo balance between these pathogens, but also the effect of these interactions and individual eradication treatments on patient outcomes. 

*S. maltophilia* is a Gram-negative pathogen of increasing significance in CF. Data from an in vitro mixed-culture biofilm model of *A. fumigatus* and *S. maltophilia* suggest an inhibitory effect of *S. maltophilia* on *A. fumigatus* growth and production of extracellular matrix [46]. Co-culture of these organisms also impacts their susceptibility to antibiotics. Susceptibility of *A. fumigatus* to amphotericin B was increased in mixed-culture biofilms, whereas *S. maltophilia* susceptibility to levofloxacin decreased [47]. These data highlight potentially clinically relevant, complex interactions between *A. fumigatus* and bacteria other than *P. aeruginosa*. Further study of interactions between *A. fumigatus* and bacteria commonly found in the CF patients is warranted. 

## 4. Treatment of ABPA with Approved Therapies

In addition to managing the symptoms of asthma or CF, treatments targeted at treating ABPA aim to prevent acute exacerbations, reduce pulmonary inflammation and to prevent progression toward end-stage fibrotic disease [48]. While there are no approved therapies for ABPA, much of our understanding of how to treat ABPA in CF patients comes from clinical trials conducted in asthmatics with ABPA. Oral corticosteroids are used in an effort to suppress inflammation and oral antifungals are used in an attempt to eradicate *Aspergillus* from the airways to reduce antigen stimulation of the allergic response [49]. Therapeutic effects are typically monitored through changes in serum IgE levels while tapering steroids until remission is observed [11,49]. Improvements in pulmonary function are a desired impact of therapy, however, deterioration of lung function in patients with APBA is variable, with some patients maintaining stable lung function and others presenting with progressive deterioration [50,51]. Current ABPA treatment paradigms have been informed by a number of clinical trials that have evaluated the effects of approved anti-inflammatory and anti-infective therapies on ABPA clinical disease (Table 1).

### 4.1. Oral Corticosteroids

The use of corticosteroids in treating ABPA in asthma has largely been based on experience in clinical practice with few randomized, controlled clinical trials studying steroid use as chronic therapy. Long-term steroid use is associated with adverse side-effects, which must be managed in parallel with the management of ABPA [12,57,58] and long-term steroid increases the risk of developing corticosteroid-dependent disease. Although studies on steroid use in patients with relapsing and chronic disease are lacking, two recent clinical studies have evaluated corticosteroid use in acute treatment naïve ABPA patients, with positive results. A comparison of high-dose and medium-dose steroid regimens in treatment-naïve ABPA patients found that both treatment protocols resulted in a similar number of acute exacerbations after 1 year and a similar number of patients with glucocorticoid-dependent ABPA after 2 years. However, the medium-dose group resulted in fewer glucocorticoid side-effects [49]. In a similar clinical study comparing prednisolone treatment to itraconazole treatment, a similar medium steroid dose resulted in high rate of clinical response and reduced IgE levels [52]. The lower steroid doses used by Agarwal et al. are similar to common treatment regimens world-wide [59]. 

### 4.2. Anti-Fungal Therapy

Use of antifungals in management of ABPA is supported by a strong biological link between *Aspergillus* infection in the airway and the resulting allergic inflammatory response that is the hallmark of ABPA inflammation. A high percentage of asthmatics sensitized to *A. fumigatus* are sputum culture-positive for *A. fumigatus* growing in their airways [6], which correlates with reduced lung function [60]. Fungal spores are largely non-inflammatory and allergic disease is primarily driven by antigens produced in the hyphal growth state [61,62,63], highlighting the fact that the germination of spores into growing hyphae is necessary for eliciting the immune response and the resulting pathophysiology of the disease (Figure 1). That these antigens are expressed in vivo and that they can be reduced by therapies that limit fungal growth is supported by several studies showing that antifungal therapy reduces *Aspergillus*-specific IgG and IgE [64]. Likewise, in a small study that examined *Aspergillus* infection in patients with ABPA and SAFS, 9 patients that were positive for *Aspergillus* infection by PCR became negative for Aspergillus infection following treatment with itraconazole. This conversion was associated with a reduction in total serum IgE [65]. 

The most common antifungal therapy used in the management of ABPA is itraconazole, a triazole that inhibits fungal cytochrome P450 synthesis of ergosterol, a critical component of the fungal cell wall [66]. Clinically, itraconazole is used to reduce fungal burden and inflammation, and also as a steroid-sparing agent to reduce the long-term usage of corticosteroids. A number of clinical studies and case series have shown the benefit of itraconazole in treating *Aspergillus* bronchitis [67] and ABPA [53,54,55,68], including ABPA patients with CF [64]. As with any anti-infective therapy, long term therapy with triazoles can lead to the emergence of resistance [69]. Of particular concern, since the predominant mechanism that azole resistance develops is through mutation of the *cyp51A* gene, the molecular target of azole activity, the development of resistance to one azole can result in broad cross-resistance to multiple azoles [70]. This concern is further underscored by the recent description of a second mechanism of multiple-azole resistance resulting from mutations in *cyp51B*, a second 14-α sterol demethylase, which may be further exacerbated by a second mutation in *hmg1* [71]. 

Oral itraconazole efficacy in asthmatics with ABPA has been studied in two randomized, placebo-controlled studies to study the clinical response and anti-inflammatory effect of treatment [53,54]. In a study of 55 asthmatics with ABPA, patients were randomized to receive oral itraconazole or placebo for 16-weeks, after which all patients received itraconazole for an additional 16 weeks in an open label extension period [54]. Itraconazole efficacy was assessed using a composite clinical response score that included reduction in corticosteroid use, reduction in IgE and either improved lung function or exercise tolerance. Compared to placebo, oral itraconazole significantly improved clinical responses and more than 70% of patients on itraconazole lowered their oral corticosteroid dose by more than 50%. In the open-label extension portion of the study 12 of the 33 patients who did not respond in the double-blind portion or were on placebo had a clinical response [54], further underscoring the efficacy of itraconazole in this patient population. 

Inflammation resulting from *A. fumigatus* antigen exposure is the main driver of clinical disease. In a second randomized, double-blind placebo-controlled study the effect of itraconazole on pulmonary inflammation was assessed in 29 subjects with stable ABPA [53]. Over 16 weeks, treatment with oral itraconazole significantly reduced the number of sputum eosinophils and eosinophil cation protein, with a significant reduction observed after only one month of therapy. Serum markers of inflammation, IgE and IgG specific to *Aspergillus* antigens, were also reduced [53]. More recently, a comparison of steroid therapy to itraconazole therapy in acute, treatment naïve patients found that while there was moderate benefit for steroid therapy over itraconazole (100% vs. 88% composite response; *p* = 0.007), itraconazole had a significant benefit to the majority of patients, with fewer side effects than steroid treatment [52]. 

Although anti-fungal drugs have not been widely studied in CF patients with ABPA, data generated in asthmatics suggests that antifungal therapy may provide benefit to CF ABPA patients. This is further supported by small studies of itraconazole in patients with CF. In a study of itraconazole in six ABPA patients, three of whom had CF, itraconazole treatment reduced steroid use and two of the three CF patients had clinical benefit, including improved lung function [68]. An additional case series of 16 CF patients with ABPA also showed that itraconazole treatment resulted in fewer acute exacerbations and provided a steroid-sparing benefit [72]. In addition to itraconazole, other available azoles such as voriconazole and posaconazole have been used with some benefit in ABPA and CF [73,74,75,76]. In one randomized trial comparing voriconazole and prednisolone, there was no difference between the two therapies after 16 weeks of dosing [55]. The opportunity to use anti-fungals in place of high dose, systemic steroids is appealing since long-term steroid use increases the risk of developing diabetes and osteoporosis, and the development of steroid-dependent ABPA is a significant concern [77,78].

Amphotericin B, a polyene anti-fungal that acts by disruption of the fungal cell wall, is commonly used as an intravenous drug to treat severe fungal infections in immunocompromised patients [79]. In an effort to directly target anti-fungal therapy to the lung, inhaled liposomal amphotericin B has been suggested as a treatment option for patients with ABPA and SAFS, however, clinical experience in small studies with inhaled amphotericin has been mixed. In one instance, inhaled amphotericin B reduced exacerbations in patients with ABPA and was reasonably tolerated after the first dose [80]. In other studies, inhaled amphotericin B has been associated with significant tolerability concerns. In a case series study of 177 patients with pulmonary aspergillosis that received inhaled amphotericin B, 66% of patients were able to tolerate an initial dose, however, 21% stopped therapy in the following 6 weeks. Only 10% of patients continued with therapy for more than 3 months, with 28% of those patients showing improvement in IgE levels [81]. Similarly, in a small clinical study of 21 adult asthmatics with SAFS and ABPA, who had failed previous antifungal therapy, 18 subjects either failed initial dosing or discontinued therapy in the following 12 months [56]. 

Elevated serum and sputum IgE levels are a hallmark of ABPA. IgE can trigger mast cell degranulation and cause hypersensitivity responses in the lung, which together drive the pathophysiology of the disease [82]. Omalizumab is an anti-IgE monoclonal antibody developed for the treatment of moderate-to-severe uncontrolled allergic asthma. Omalizumab has been used off label in a series of small studies in adults and children with CF and ABPA. In several case reports, omalizumab treatment has shown promise with improved lung function, reduced steroid use and fewer exacerbations in CF patients [83,84,85]. However, not all studies have shown efficacy with omalizumab [86], and the only well controlled randomized clinical trial was terminated early due to poor enrollment (NCT00787917). Further study is warranted for this approach.

## 5. New Therapies to Treat ABPA and Fungal Infections

Despite the advances in diagnosis and management of ABPA, there remains a significant unmet medical need for the treatment of ABPA. The primary antifungal therapy, oral itraconazole, is generally safe and well tolerated in both CF and non-CF patients, though there is an extensive list of drug-drug interactions (DDI), which requires drug monitoring during therapy. Itraconazole absorption and pharmacokinetics can be highly variable, resulting in inconsistent exposure across patients, which may impact the consistency of clinical responses [68,72]. In healthy volunteers, oral bioavailability of itraconazole is 55% and is impacted by digestive function [87]. In CF patients, itraconazole exposure is variable and a significant fraction of patients may not achieve therapeutic dose levels. In a study of 11 CF patients, oral itraconazole dosing resulted in sub-therapeutic plasma concentrations in 5 of 11 patients. Low plasma concentrations correlated with variable sputum itraconazole concentrations that were below the reported minimum inhibitor concentrations of itraconazole against *A. fumigatus* [88]. Variable itraconazole pharmacokinetics following oral dosing highlight the challenge of achieving the high, and consistent lung exposure required for efficacy. Consistent with this, a clinical study showing no clinical benefit of itraconazole in CF patients also found that most patients failed to adequate itraconazole exposure [89]. 

Given the challenges of oral anti-fungal therapy, efforts have aimed to improve the bioavailability of itraconazole to increase exposure in the lungs. One recent approach has been the development of SUBA-itraconazole, an oral formulation with improved pharmacokinetics that rapidly achieves therapeutic levels in the lung [90]. Alternatively, several groups have aimed to use inhalation as an approach to directly deliver anti-fungal agents to the site of disease. Inhalation offers the potential to overcome many of the challenges of oral therapies, including achieving high and consistent drug concentrations at the site of infection. Achieving high drug concentrations may limit the emergence of drug-resistant strains or alternatively enable the treatment of drug-resistant infections by achieving concentrations in excess of minimum fungicidal concentrations. Several of these inhalation approaches utilize novel drug delivery technologies to reformulate itraconazole or voriconazole for delivery to the lungs, thereby leveraging the known activity of azoles against *A. fumigatus*. In addition, PC945, a novel azole delivered by liquid nebulization is also in development as a therapy for treating pulmonary fungal infections (Table 2). 

## 6. PUR1900: Inhaled Itraconazole

PUR1900 (Pulmatrix Inc, Lexington, MA, USA) is a dry powder formulation of itraconazole being developed using a proprietary inhaled delivery technology called iSPERSE [91]. A Phase 1 study in healthy volunteers and adult asthmatic patients (NCT03479411) demonstrated that PUR1900 was safe and well-tolerated. Compared to oral dosing, PUR1900 achieved higher lung and lower plasma itraconazole exposure relative to oral itraconazole treatment [92]. After a single dose of inhaled PUR1900 in asthmatics, therapeutic itraconazole sputum concentrations were observed for over 24 hours in most patients [92].

### 6.1. Inhaled Voriconazole

ZP-059 (Zambon, Milan, Italy) is a dry powder formulation of voriconazole being developed using a novel spray drying technology for the treatment of ABPA in asthma [93]. This formulation was recently evaluated in a Phase 1 study (NCT04229303); however, no results have been reported to date.

TFF-VORI (TFF Pharmaceuticals, Austin, TX, USA) is a dry powder formulation of voriconazole formulated using thin film freezing technology, which produces excipient-free nanoaggregates of drug for inhalation [94,95]. A Phase 1b clinical safety, tolerability and pharmacokinetic study in adults with asthma (NCT04576325) began in late 2020 with an estimated completion date of December 2021. 

### 6.2. PC945: A Novel Inhaled Azole

PC945 (Pulmocide, London, UK) is a novel triazole being developed for liquid nebulization for the treatment of IPA, with potential for use in ABPA. PC945 is a potent inhibitor of ergosterol synthesis, exhibiting 14-fold greater potency than voriconazole and 2.6-fold more potency than posaconazole against *A. fumigatus* [96]. A Phase 1 study in healthy volunteers and adult asthmatic patients (NCT02715570) showed that following inhalation, PC945 was slowly absorbed from the lung and led to low systemic exposure, suggesting an improved safety and DDI profile relative to oral itraconazole [97]. 

## 7. Conclusions

Significant advances have been made in understanding the incidence and severity of *Aspergillus*-related allergic diseases in patients with CF. With this understanding, more work is needed to decipher the relationship between *A. fumigatus* infections and different clinical outcomes. An increased appreciation of the clinical significance of ABPA has led to an understanding of the importance of the interactions between fungal and bacterial infections. Additional research in these areas is warranted to further characterize the complex microbial ecology of the CF lung and to help identify new treatment strategies for the management of disease. In recent years there have been several large, well-controlled clinical studies of therapies for ABPA, which have significantly improved treatments for patients and established a framework for the continued study of new therapies in development. The assessment of anti-fungal drugs with novel mechanisms of action as treatments for ABPA and other allergic fungal diseases would be a welcome step towards improving patient lives. 

## Figures and Tables

**Table 1:** Randomized, controlled clinical trials conducted in ABPA.

Drug	Dose	Design	N	Duration	Primary Outcome	Reference
Prednisolone	0.5mg/kg * 0.75mg/kg *	Randomized, controlled	92	6 to 8 weeks followed by taper for up to 10 months	Exacerbation rate Steroid-dependent ABPA	[49]
Itraconazole Prednisolone	200mg BID 0.5mg/kg *	Randomized, controlled	131	16 weeks	Composite clinical response Decline in IgE Exacerbation rate	[52]
Itraconazole	400mg QD	Randomized, double blind, placebo controlled	29	16 weeks	Sputum eosinophil count	[53]
Itraconazole	200mg BID	Randomized, double blind, placebo controlled	55	16 weeks	Composite clinical response	[54]
Voriconazole Prednisolone	200mg BID 0.5mg/kg *	Randomized, controlled, unblinded	50	16 weeks	Composite clinical response Exacerbation rate	[55]
Inhaled amphotericin B	10mg BID	Randomized, controlled	21	16 weeks	Time to first exacerbation	[56]
Omalizumab	600 mg	Randomized, double blind, placebo controlled	14 **	24 weeks	Requirement for rescue corticosteroids	NCT00787917

* Starting doses, regimens involved a pre-specified reduction in dose and tapering regimen; ** Discontinued due to poor enrollment.

**Table 2:** Novel drugs in development as treatments of ABPA.

Product	Company	Formulation	Drug	Clinical Trials	Primary Indication	Development Phase
PUR1900	Pulmatrix	Dry Powder	Itraconazole	NCT03479411 NCT03960606	ABPA	Phase 2
ZP-059	Zambon	Dry Powder	Voriconazole	NCT04229303	IPA	Phase 1
TFF-Vori	TFF	Dry Powder	Voriconazole	NCT04576325	ABPA	Phase 1
PC945	Pulmocide	Liquid Nebulization	Novel Azole	NCT02715570	IPA	Phase 1
